# Supplementary material for: Prospective Longitudinal Study of Putative Agents Involved in Complex Gill Disorder in Atlantic salmon (Salmo salar)
Source: Pathogens. 2022 Aug 3;11(8):878. doi: 10.3390/pathogens11080878 (PMC9415954; doi:10.3390/pathogens11080878)
Supplement: Supplementary file 1 [file pathogens-11-00878-s001.zip › pathogens-1755070-supplementary.pdf]

Supplementary Table S1. Average and standard deviation (sd) of the environmental parameters measured 14 days before the sampling point.

	Oxygen Saturation (%)				Salinity (ppt)				Water Temperature (°C)			
	Farm A		Farm B		Farm A		Farm B		Farm A		Farm B	
Week	Average	sd	Average	sd	Average	sd	Average	sd	Average	sd	Average	sd
6	95.3	2.3	-	-	30.6	1.9	-	-	8.3	0.4	-	-
10	103.4	6.4	97.4	4.4	30.9	3.1	34.0	0.0	7.6	0.4	7.4	0.2
14	103.8	3.6	98.2	4.9	32.9	1.4	34.0	0.0	8.2	0.3	7.9	0.3
19	104.1	1.3	113.4	6.9	32.3	2.2	34.0	0.0	10.7	0.5	12.1	0.9
23	97.1	7.6	114.1	4.1	34.6	0.9	34.0	0.0	11.3	0.3	10.8	0.5
28	90.4	6.0	107.1	5.7	32.4	1.2	34.0	0.0	13.0	0.4	12.8	0.4
30	93.5	5.0	96.9	6.3	28.3	3.5	34.0	0.0	13.3	0.2	12.8	0.8
32	88.1	8.0	96.4	6.2	30.1	3.0	34.0	0.0	13.6	0.2	12.8	0.4
34	89.3	4.5	94.0	5.2	30.9	3.0	34.0	0.0	13.7	0.2	12.9	0.3
36	83.0	3.3	96.7	5.3	31.6	3.4	34.0	0.0	13.2	0.4	12.4	0.2
38	80.2	2.6	89.0	3.7	27.2	2.0	34.0	0.0	12.3	0.4	12.3	0.3
40	85.0	3.7	88.5	3.6	29.1	2.6	34.0	0.0	12.0	0.5	11.8	0.2
43	81.9	2.5	91.3	3.2	31.1	1.9	34.0	0.0	11.0	1.0	10.9	0.5
45	83.1	2.1	89.8	5.4	32.0	1.2	34.0	0.0	10.9	0.4	10.3	0.3
47	86.9	3.5	88.7	4.4	31.3	1.3	34.0	0.0	10.7	0.6	10.5	0.6
49	88.5	1.8	93.1	4.2	30.6	2.6	34.0	0.0	9.3	0.6	9.7	0.4
52	86.2	1.5	90.4	1.9	31.2	1.1	34.0	0.0	9.0	0.4	9.0	0.2
54	82.4	12.5	90.3	2.8	32.4	1.7	34.0	0.0	9.0	0.4	9.0	0.1
57	90.1	0.7	92.3	3.3	33.4	14.6	34.0	0.0	8.2	0.3	7.9	0.3

Supplementary Table S2. Comparison of the GAMs for the prediction of Ct value for different pathogens (*D. lepeophtherii*, *N. perurans*, SGPV and *Ca*. B. cysticola) across weeks and between farms. Note that Model 3 always gave the lowest AIC results.

	AIC value of the model			
Pathogen	Model 0	Model 1	Model 2	Model 3
D. lepeophtherii	1470.037	1286.769	1166.898	1120.162
N. perurans	1463.528	1369.372	1464.715	1221.686
SGPV	1113.753	1067.951	1039.969	1038.828
Ca. B. cysticola	1189.390	1109.727	1103.921	1101.267

Supplementary Table S3. Criteria for the histological gill scoring system used in this study. Slightly modified from Mitchell et al. (2012).

Score	Lamellar Epithelium Hyperplasia	Lamellar Fusion	Cellular Death	Circulatory Disturbances	Inflammation
None (0)	None or very minor	None or very minor	None or very minor	None or very minor	None or very minor
Mild (1)	Mild increase in lamellar epithelial cell (<10% of gill tissue affected)	Occasional focal fusion of filaments (<10% of gill tissue affected)	Scattered, occasional, degenerating necrotic or apoptotic cells and/or cell sloughing (<10% of gill tissue affected)	Scattered, occasional vascular changes (<10% of gill tissue affected)	Scattered, occasional inflammatory cells (<10% of gill tissue affected)
Moderate (2)	Moderate multifocal or widespread increase in lamellar epithelial cells, affecting 10–50% of the tissue	Multifocal areas of fusion, affecting 10–50% of gill tissue interspersed with normal gill tissue	Multifocal, degenerating necrotic or apoptotic cells and/or cell sloughing affecting 10–50% of the tissue	Vascular changes in multifocal areas, affecting 10–50% of the tissue	Inflammatory cells in multifocal areas, affecting 10–50% of the tissue
Severe (3)	Extensive multifocal or widespread increase in lamellar epithelial cells, affecting >50% of the tissue	Extensive fusion and loss of normal architecture, affecting >50% of the tissue	Extensive, degenerating necrotic or apoptotic cells and/or cell sloughing affecting >50% of the tissue	Multifocal to widespread vascular changes, affecting >50% of the tissue	Multifocal to widespread inflammatory cells, affecting >50% of the tissue
Absence (0) or presence (1)	• Lamellar tissue disruption (disruption of a group of lamellae, associated with haemorrhages and cell death)<br>• Lamellar oedema (≥10% of gill tissue affected)<br>• Eosinophilic Granular Cells (increase of EGCs numbers within the filaments)<br>• Bacteria- Epitheliocysts (variable sized basophilic inclusion bodies found mainly in the branchial epithelium)<br>• Bacteria- *Tenacibaculum* spp.<br>• Protists - *Neoparamoeba* spp.<br>• Protists - *Ichthyobodo* spp. (Costia)<br>• Protists - *Trichodina* spp.<br>• Unidentified metazoan organisms (i.e. filamental metacercaria)				
